# Thought probes during prospective memory encoding: Evidence for perfunctory processes

**DOI:** 10.1371/journal.pone.0198646

**Published:** 2018-06-06

**Authors:** Michael K. Scullin, Mark A. McDaniel, Michelle N. Dasse, Ji hae Lee, Courtney A. Kurinec, Claudina Tami, Madison L. Krueger

**Affiliations:** 1 Baylor University, Department of Psychology & Neuroscience, Waco, TX, United States of America; 2 Washington University in St. Louis, Department of Psychological and Brain Sciences, St. Louis, MO, United States of America; University College London, UNITED KINGDOM

## Abstract

For nearly 50 years, psychologists have studied prospective memory, or the ability to execute delayed intentions. Yet, there remains a gap in understanding as to whether initial encoding of the intention must be elaborative and strategic, or whether some components of successful encoding can occur in a perfunctory, transient manner. In eight studies (N = 680), we instructed participants to remember to press the *Q* key if they saw words representing fruits (cue) during an ongoing lexical decision task. They then typed what they were thinking and responded whether they encoded fruits as a general category, as specific exemplars, or hardly thought about it at all. Consistent with the perfunctory view, participants often reported mind wandering (42.9%) and hardly thinking about the prospective memory task (22.5%). Even though participants were given a general category cue, many participants generated specific category exemplars (34.5%). Bayesian analyses of encoding durations indicated that specific exemplars came to mind in a perfunctory manner rather than via strategic, elaborative mechanisms. Few participants correctly guessed the research hypotheses and changing from fruit category cues to initial-letter cues eliminated reports of specific exemplar generation, thereby arguing against demand characteristics in the thought probe procedure. In a final experiment, encoding duration was unrelated to prospective memory performance; however, specific-exemplar encoders outperformed general-category encoders with no ongoing task monitoring costs. Our findings reveal substantial variability in intention encoding, and demonstrate that some components of prospective memory encoding can be done “in passing.”

## Introduction

Prospective memory is an umbrella term that refers to remembering to execute goals, intentions, and chores in the future [1,2]. A prototypical prospective memory task is remembering to pick up milk at the grocery store, or, remembering to go to the grocery store at all. However, prospective memory encompasses a broader array of relationship-oriented tasks (e.g., returning a friend’s text message), household chores (e.g., take out the trash), health-oriented intentions (e.g., adhering to medication schedules), society-oriented goals (e.g., identifying missing or wanted persons), and workplace tasks and routines [3–5]. The goal of the present work was to advance understanding of how intentions are encoded.

### Encoding processes in prospective memory

An intuitive view of prospective memory encoding is that intention formation is deliberate, elaborative, and strategic. Consider, for example, the Theory of Planned Behavior, which states that intention formation is the “*conscious* plan or decision to *exert effort* to enact the behavior” (p. 1430 [6]; italics added). The more individuals draw upon working memory resources at encoding, the more likely they are to successfully complete their planned intention [7–10]. Furthermore, when studying word lists for later recognition or recall (“retrospective memory”), devoting greater working memory resources toward elaborative or organizational processing increases the probability of those items being retained [11–13]. Therefore, according to one view, the successful encoding of prospective memories will require strategic, controlled processes to elaborate on the intention (e.g., generating many retrieval cues). For convenience, we label this general position as the *strategic/elaborative* encoding view.

On the other hand, some information might be encoded quickly and with minimal cognitive effort, such as the associations amongst studied items [14–16]. According to this literature, it is plausible that some aspects of prospective memory encoding may be accomplished “in passing.” Anecdotally, one might remember to purchase several specific ingredients for a chicken curry dinner when only consciously encoding “curry dinner” as a general category (this specific example assumes the absence of strategic retrieval mode processes when arriving at the grocery store). Some researchers argue that prospective memory encoding can even be implicit, such as when one remembers to turn on their phone after a colloquium (after politely turning it off to listen), or when one remembers to resume drafting an e-mail after being interrupted by a phone call [17,18]. This general orientation anticipates that some components of prospective memory encoding may be cursory, transient, implicit, or otherwise engage minimal working memory resources. We label this position as the *perfunctory/transient* encoding view.

### Encoding manipulations in prospective memory experiments

Some prospective memory research favors the strategic/elaborative encoding view [19,20]. When participants use an encoding strategy, they tend to generate more retrieval cues and perform better on tests of prospective memory [21–24]. In addition, neuroimaging studies suggest that greater activation during encoding (e.g., in motor regions) may predict better later retrieval [25–27]. Furthermore, when young and older adults encode complex prospective memory tasks, the older adults tend to show deficits in plan formation, possibly due to an age-related deficit in working memory resources [28].

However, not all studies have observed age differences in prospective memory planning [29] or that greater neural activation during encoding predicts later retrieval [30]. Strategic planning often diverges from prospective memory execution [31], and less elaborative planning can sometimes lead to better prospective memory [28]. Some intentions may even be implicitly formed, such as the intention to later put a wristwatch back on after being told to put it away; in observing that many participants could complete this implicit wristwatch task, Kvavilashvili and colleagues [18] concluded that “the conscious formation of intention may not always be necessary for successful remembering as stipulated in the prospective memory literature” (p. 873). To be clear, most prospective memory laboratory paradigms encourage, if not require, that the intention is consciously encoded. Whether some components of prospective memory encoding can still be perfunctory, even in a controlled laboratory environment, remains under-studied.

### Overview of the current work

**Fig 1:**
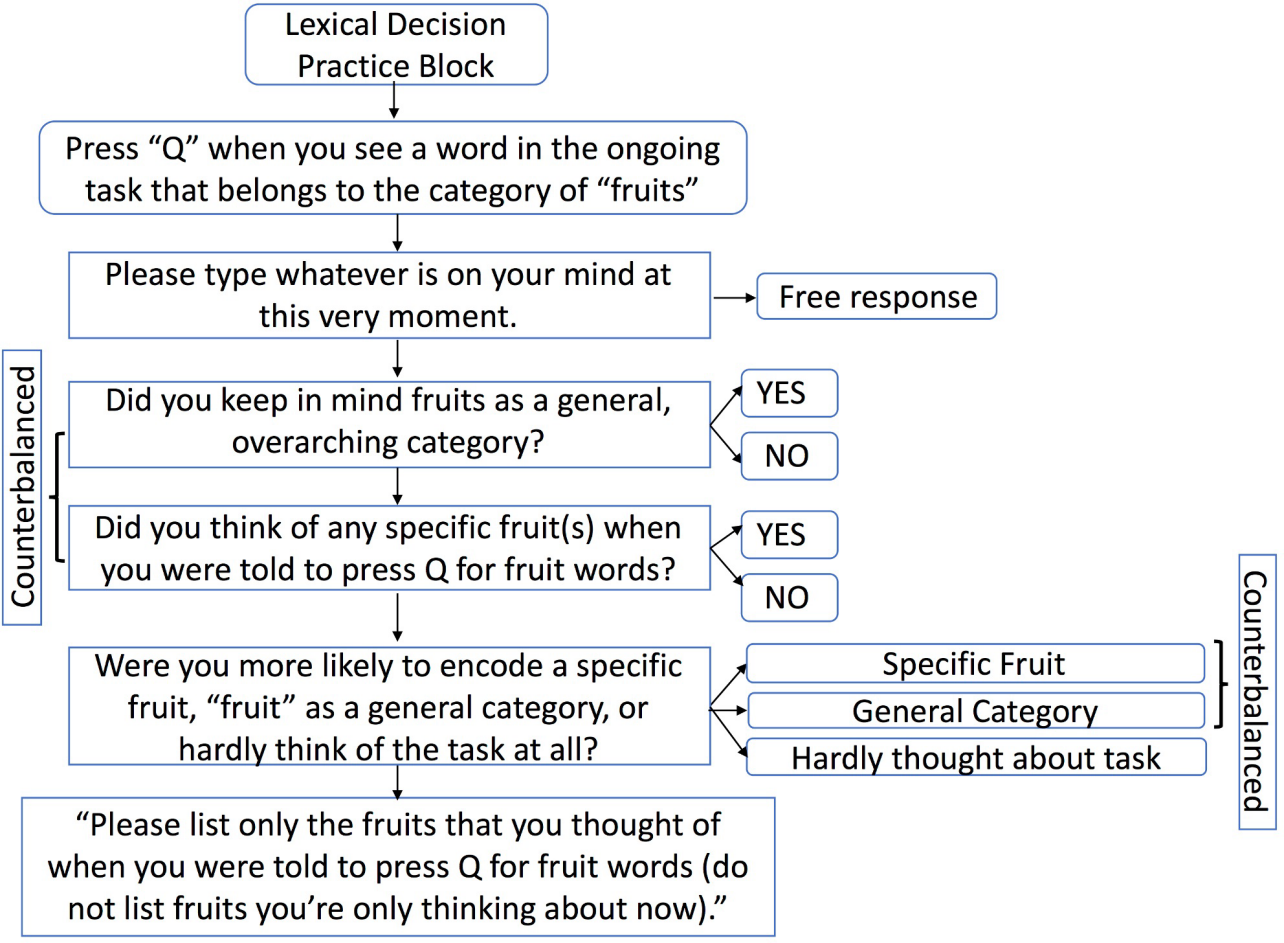
Depiction of the encoding thought probe procedure. In Experiments 1–2, the target category was animals. This figure was adapted with permission [24].

**Fig 2:**
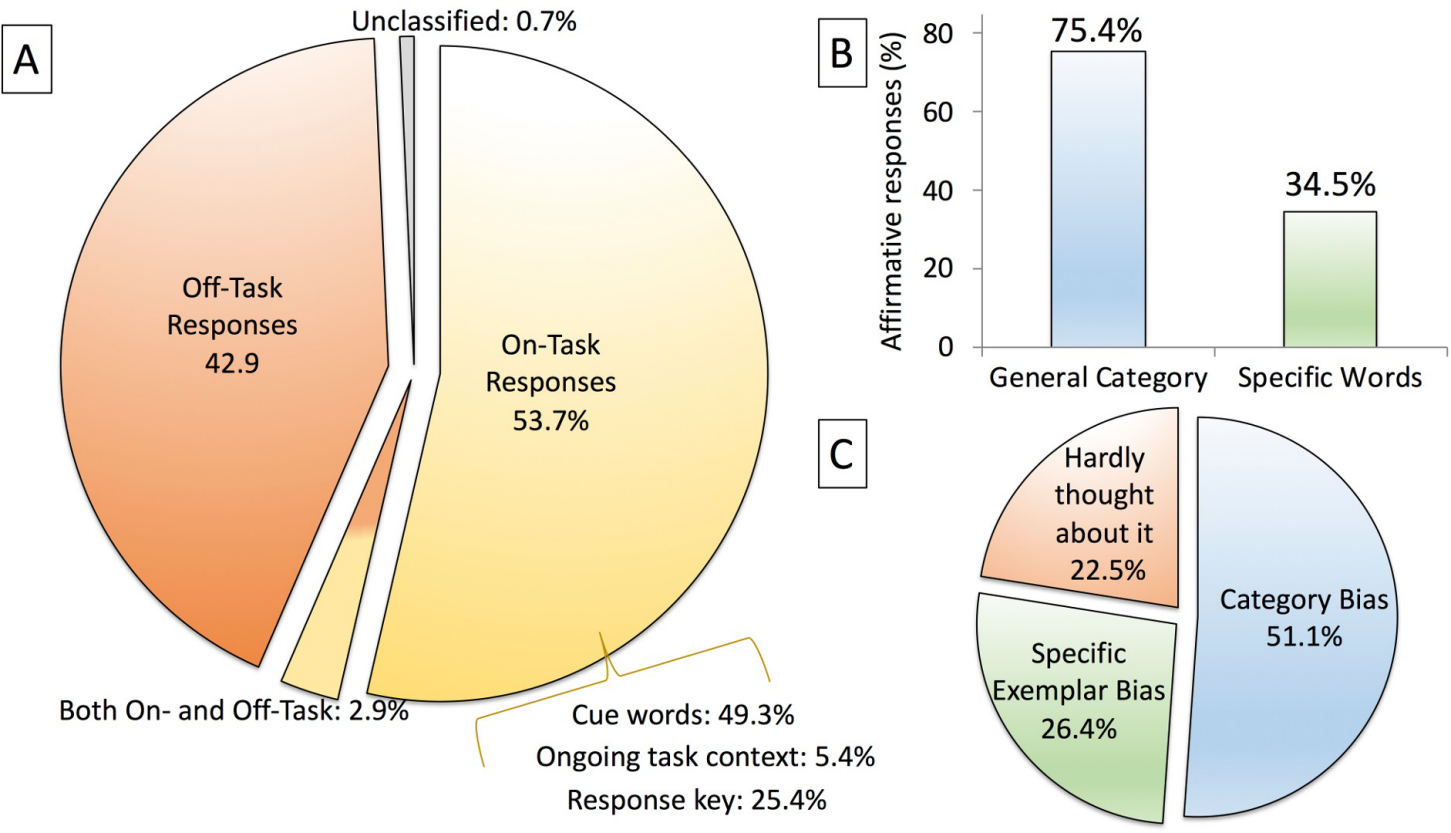
Encoding thought probe data collapsed across Experiments 1–8. The figures depict the aggregate (A) free response data, (B) generation of specific exemplars, and (C) bias toward different encoding strategies.

Across eight experiments, we used thought probes to gauge the processes operating during intention formation. There are many laboratory procedures for studying prospective memory, but the most common approach is the Einstein-McDaniel paradigm [32]. As shown in Fig 1, participants practiced an ongoing task (lexical decision) and then were instructed to remember to press a specific key (*Q*) in response to a target stimulus (e.g., animal words). Immediately after encoding, participants reported what was currently on their mind and responded to questions targeted at identifying encoding processes. The encoding thought probe approach complements previous work that used thought probes during retrieval [33–35] as well as studies that inferred encoding processes from verbal plan descriptions, neuroimaging outcomes, later retrieval/performance, and simulations [21,25,28,36]. Given the number of experiments included, we summarize the research questions and results in Table 1 and Fig 2. In overview, Experiments 1–7 were designed to address basic science questions about the processes operating at encoding. Experiment 8 was designed to test the consequences of these encoding processes for prospective memory retrieval.

**Table 1:** A summary of the research questions and main results/interpretations across eight studies/experiments of prospective memory encoding. The reader is directed to the methods and results section of each study for research details and inferential statistics.

Experiment	Research Questions	Main Findings
Experiment 1	• What is on participants’ minds during intention encoding?	• Approximately half of participants mind wander during encoding.
Experiment 2	• Do prime words affect encoding?	• Prime words affect which specific cues are encoded.
Experiment 3	• Is encoding strategic or perfunctory?	• Specific-cue encodings occur in a perfunctory manner.
Experiment 4	• Do older adults show less specific encoding than young adults?	• No age differences, which is consistent with the perfunctory view.
Experiment 5	• Are participants aware of the research hypotheses on encoding?	• No, demand characteristics do not explain perfunctory-encoding results.
Experiment 6	• Does a verbal report of the instructions to the experimenter eliminate mind wandering during encoding?	• No, many encodings remain perfunctory even with a verbal “experimenter check.”
Experiment 7	• How do alterations in the prospective memory cue affect encoding?	• Encoding is perfunctory for categorical cues and strategic for syllable cues. • For initial-letter cues, participants do not generate specific examples.
Experiment 8	• Do encoding processes predict later prospective memory performance? • Do encoding processes affect reliance on monitoring versus spontaneous retrieval? • Will perfunctory encodings still allow for later retrieval?	• Yes, specifically encoded intentions led to better performance. • Yes, specifically encoded intentions led to reduced (no) monitoring costs. • Yes, perfunctory encodings can still lead to successful performance.

## Experiments 1–3

We investigated encoding processes by using categorical cues (animals, fruits [37]). One view is that participants will encode the prospective memory task exactly as the experimenter instructs them to: as a general, superordinate category [38]. An alternative view is that participants will generate specific category exemplars, such as apple [39]. If participants generate specific exemplars, then the critical theoretical question is whether they do so in a strategic/elaborative manner (as in category fluency neuropsychological tests [40]), or whether they generate exemplars “in passing” (e.g., via spreading activation in semantic networks [41,42]). To test whether we could bolster the exemplar-generation process, some participants were shown a prime word (e.g., apple) during a practice block.

### Method

#### Participants

Washington University undergraduate students (*N* = 68 in Experiment 1 and *N* = 61 in Experiment 2) and Baylor University undergraduate students (*N* = 68 in Experiment 3) participated for partial class credit in the present protocol as well as an unrelated protocol on juror decision making. The unrelated protocol contained no animal or fruit stimuli and participants were told that they would perform a series of cognitive tasks (i.e., all procedures were described in one informed consent). Nevertheless, we ensured the generality of our findings in Experiments 4, 5, 6, and 8 by conducting the prospective memory procedures without an unrelated protocol. Table 1 foreshadows that the critical findings on perfunctory/transient processes replicated. Note that, in Experiment 2, one participant was excluded for inadvertently being run using an incorrect program (N = 60).

All experiments presented in this manuscript were approved by the local IRB (Baylor University, Washington University) and all participants provided written consent prior to participating. E-Prime 2.0 files and data are available at Open Science Framework (osf.io/63a7f).

#### Procedure

As shown in Fig 1, and following previous research [24], participants first learned the lexical decision task instructions (referred to as the word/nonword task) to respond as quickly and accurately as possible whether a string of letters formed a word or not (by pressing keys marked “Y” and “N” on the number pad). Then they practiced the lexical decision task for 10 trials, during which they received speed and accuracy feedback following each trial. The prime word *fish* was presented during the practice block in Experiment 1, but not in Experiment 2. In Experiment 3, we randomly assigned participants to prime and no-prime conditions that differed in whether the word *apple* was presented during the practice block (cf. [43]).

Participants were next given the following prospective memory task instructions (modifications for Experiment 3 are provided in brackets):

“In this experiment, we are also interested in your ability to remember to perform an action at a given point in the future. Therefore, during the word/nonword task, we would like you to perform a special action whenever you see a word that belongs to the category ANIMAL [FRUITS]. Whenever you see an animal [a fruit] word, you should remember to press the 'Q' key. Press Q to continue.”

On the next screen, participants typed whatever was on their mind at that moment, and then asked two yes/no questions about encoding specific examples of animals (fruits) versus keeping animals (fruits) in mind as a general, overarching category (order counterbalanced). They were further asked whether they were more focused on encoding specific examples, the general category, or if they hardly thought about this task at all (list order counterbalanced for specific/general options). Lastly, if participants previously indicated that they generated specific examples, they were asked to type which examples they thought of when they encoded the prospective memory task (and to avoid typing any new examples they just thought of). We used this thought probe procedure in every experiment, with the exception that in Experiment 1 participants were only asked to type what was on their mind, whether they thought of any specific animal words, and (if so) which animal words they encoded.

#### Statistical analysis

To classify the free responses, three members of the research team independently rated the responses as “on-task,” “off-task,” or “both on and off task” [24]. They next rated the “on-task” responses according to whether they mentioned the target cue type, the ongoing task (contextual processing [44]), and the response key (motor planning [45]). The three raters were masked to experimental conditions and met to resolve any disagreements. In every experiment, ≥98% of the responses were reconcilable after discussion, and the remaining responses were listed as “unclassifiable.”

We conducted chi-square tests to determine whether there were significant differences in the distribution of encoding responses. Where a cell value was <5, we used Yates’ [46] correction. We also tested whether order counterbalance affected responses to the yes/no or encoding bias questions. In Experiment 3, we used *t*-tests to determine whether encoding durations (reading time on the encoding instructions screen) were associated with encoding thought probe responses (encoding duration data were not recorded by e-prime in the first two experiments).

### Results

#### On-mind free responses

The free response data are presented in Table 2 and aggregated across all experiments in Fig 2. We predicted that because the prospective memory procedure was brief and the encoding instructions are one of the most critical elements in prospective memory studies, that nearly all free responses would include on-task, experiment-relevant content. This prediction was clearly disfavored as there was a similar frequency of solely on-task and solely off-task responses (Experiment 1: χ^2^ < 1; Experiment 2: χ^2^(1) = 1.21, *p* = .27; Experiment 3: χ^2^ < 1). Participants’ thoughts often focused on food (“biscuits”), sleep (“I’m sleepy”), class (“I have an exam tomorrow”), relationships (“ex-boyfriend problems”), and current events (“world series win”). Table 2 further demonstrates that most on-task comments focused on the prospective memory cue type, with fewer encoding processes related to motor planning and very few to contextual processing.

**Table 2:** Free response data classification as on-task (task-related) or off-task (task unrelated) across experiments. On-task responses were further classified as mentioning the ongoing task (context), prospective memory response key, or cue words. The on-task specification numbers will not sum to 100% due to some participants providing only miscellaneous responses (e.g., “this experiment”) and others listing multiple components (e.g., response key and cue words).

			On- Versus Off-Task Classification				On-Task Specifications		
Experiment (Condition)	PM Cue Type	N	On-Task	Off-Task	On- and Off -Task	Cannot Classify	Ongoing Task (LDT)	PM Response Key (Q)	PM Cue Words
Experiment 1 (Fish Prime)	Animal Category	68	48.5%	44.1%	5.9%	1.5%	13.5%	32.4%	51.4%
Experiment 2 (No-Prime)	Animal Category	60	50.0%	40.0%	8.3%	1.7%	2.9%	20.0%	54.3%
Experiment 3 (No-Prime)	Fruit Category	35	45.7%	51.4%	2.9%	0%	0%	5.9%	58.8%
Experiment 3 (Apple Prime)	Fruit Category	33	57.6%	39.4%	3.0%	0%	15.0%	35.0%	60.0%
Experiment 4 (young adults)	Fruit Category	55	52.7%	38.2%	9.1%	0%	2.9%	29.4%	35.3%
Experiment 4 (older adults)	Fruit Category	60	66.7%	31.7%	0%	1.7%	4.9%	53.7%	51.2%
Experiment 5 (PARH)	Fruit Category	59	41.1%	58.9%	1.8%	0%	3.8%	34.6%	50.0%
Experiment 6 (Verbal Check)	Fruit Category	62	53.2%	46.8%	0%	0%	0%	14.5%	51.6%
Experiment 7 (Category)	Fruit Category	33	54.5%	45.5%	0%	0%	11.1%	5.6%	61.1%
Experiment 7 (Initial-letter)	Initial Letter *t*	34	38.2%	58.8%	0%	2.9%	15.4%	38.5%	30.8%
Experiment 7 (Syllable)	Syllable *tor*	32	46.9%	53.1%	0%	0%	0%	26.7%	33.3%
Experiment 8 (No-Prime)	Fruit Category	89	70.8%	28.1%	1.1%	0%	3.1%	10.9%	48.4%

Abbreviations: LDT: Lexical Decision Task; PARH: Perceived Awareness of the Research Hypothesis scale; PM = prospective memory

#### Yes/no question responses

We next investigated the quality of encoding as the proportion of participants responding affirmative to general category encoding and specific exemplar encoding. The data are included in Table 3 and illustrated collapsed across all experiments in Fig 2. Contrary to the view that participants never generate specific exemplars during categorical cue tasks, a significant proportion of participants reported to generating specific exemplars of animals/fruits at encoding in Experiment 1 (χ^2^(1) = 22.53, *p* < .001, Yates’ correction), Experiment 2 (χ^2^(1) = 20.26, *p* < .001, Yates’ correction), and Experiment 3 (χ^2^(1) = 37.60, *p* < .001, Yates’ correction). When participants were forced to choose whether they focused more on general category encoding or specific exemplar encoding, participants indicated a general category bias in Experiment 2 (χ^2^(1) = 16.81, *p* < .001), but not in Experiment 3 (χ^2^(1) = 2.69, *p* = .10).

**Table 3:** Frequency of responses to yes/no and encoding bias questions across experiments.

Experiment (Condition)	Cue Type	N	General–Yes %	Specific–Yes %	Category Bias %	Specific Bias %	Didn’t think about PM %
Experiment 1 (Fish Prime)	Animal Category	68	n/a	30.9%	n/a	n/a	n/a
Experiment 2 (No-Prime)	Animal Category	60	78.3%	31.7%	58.3%	21.7%	20.0%
Experiment 3 (No-Prime)	Fruit Category	35	65.7%	40.0%	42.9%	25.7%	31.4%
Experiment 3 (Apple Prime)	Fruit Category	33	81.8%	51.5%	36.4%	27.3%	36.4%
Experiment 4 (young adults)	Fruit Category	55	89.1%	30.9%	61.8%	27.3%	10.9%
Experiment 4 (older adults)	Fruit Category	60	80.0%	31.7%	61.7%	26.7%	11.7%
Experiment 5 (PARH)	Fruit Category	59	86.4%	39.0%	55.9%	32.2%	11.9%
Experiment 6 (Verbal Check)	Fruit Category	62	72.6%	45.2%	58.1%	29.0%	12.9%
Experiment 7 (Category)	Fruit Category	33	75.8%	33.3%	36.4%	36.4%	27.3%
Experiment 7 (Initial-letter)	Initial Letter *t*	34	47.1%	11.8%	41.2%	11.8%	47.1%
Experiment 7 (Syllable)	Syllable *tor*	32	53.1%	25.0%	34.4%	28.1%	37.5%
Experiment 8 (No-Prime)	Fruit Category	89	76.4%	37.1%	48.3%	24.7%	27.0%

Abbreviations: PM = prospective memory; PARH: Perceived Awareness of the Research Hypothesis scale; n/a indicates that the question was not included in the procedure.

#### Frequency of cue words generated (priming effects)

When *fish* was a prime during a practice block (Experiment 1), it was the most commonly mentioned cue word (*n* = 10); when it was not primed (Experiment 2), no participants reported encoding *fish*, χ^2^(1) = 7.64, *p* = .006 (Yates’ correction). In Experiment 3, *apple* was the most frequently generated fruit word in both the *no-prime* condition (*n* = 9) and the prime condition (*n* = 15), χ^2^(1) = 2.90, *p* = .09. Perhaps the magnitude of the priming effect depends on how typical the exemplar is to the encoded category (e.g., *fish* is a less typical exemplar of animals than *apple* is of fruits [47]).

#### Encoding duration

**Fig 3:**
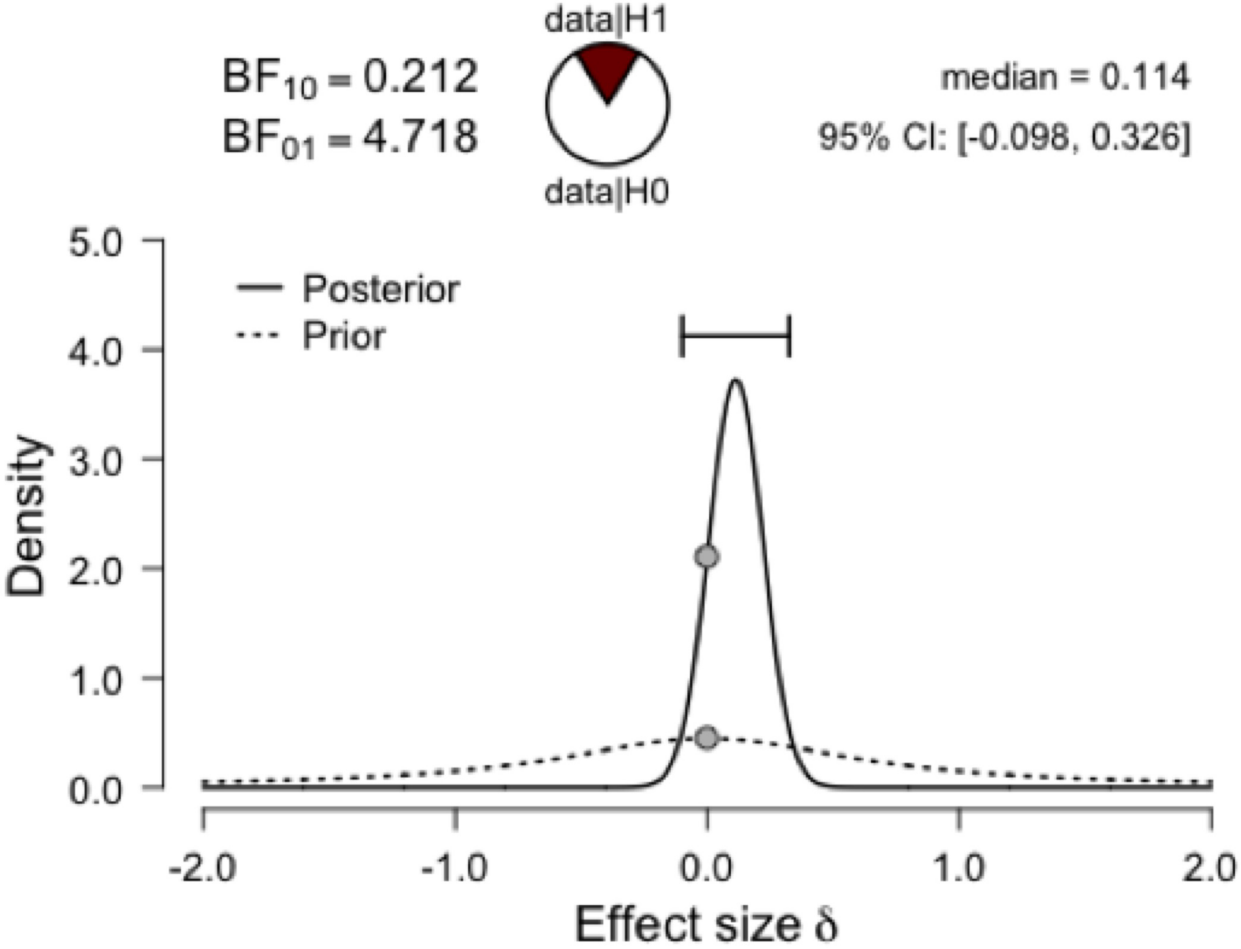
Encoding response time data relative to specific exemplar generation across experiments. The figure displays the prior and posterior distributions for effect size δ as a function of generation of specific exemplars. The sample size was limited to young adults in categorical prospective memory conditions. The BF_01_ and BF_10_ values from the Bayesian t-test both showed substantial evidence for the null hypothesis that encoding duration was similar for individuals who generated specific exemplars (n = 136; *M* = 21.62 sec, *SD* = 7.93) as those who did not generate exemplars (n = 212, *M* = 22.86 sec, *SD* = 11.76). The figures were produced using JASP software [56].

If specific exemplar generation is the result of a strategic/elaborative encoding process, then encoding durations should be greater for individuals who reported having generated specific exemplars [48]. By contrast, Table 4 shows that there was no association between encoding duration and the likelihood of generating a specific exemplar, even when selecting only individuals who were not mind wandering (r(35) = -.17, *p =* .32). Fig 3 builds on this encoding duration null finding by presenting Bayesian prior and posterior distributions for effect size δ for Experiments 3–8. Collapsed across all studies, there was substantial evidence in favor of the null hypothesis that encoding duration was unrelated to specific exemplar generation (BF_10_ = 0.21). Thus, exemplar generation seems perfunctory, perhaps the result of automatic, spreading activation processes [42,49,50].

**Table 4:** Encoding duration data (in seconds) across Experiments 3–8. Encoding duration data were not collected in Experiments 1–2. Positive correlations indicate that longer encoding durations were associated with more specific exemplar generation and more mind wandering.

Experiment (condition)	N	Mean ± SD	Range	Skewness	Kurtosis	*r—*specific exemplar	*r–* off task
Experiment 3	68	19.61 ± 5.94	3.24–42.50	0.87	4.12	*r* = .14, *p* = .25	*r* = -.32, *p* = .008*
Experiment 4 (young adults)	48^†^	22.73 ± 17.48	3.31–91.85	2.75	8.78	*r* = .07, *p* = .62	*r* = -.01, *p* = .95
Experiment 4 (older adults)	57^†^	22.06 ± 12.03	4.70–62.54	1.64	2.61	*r* = -.19, *p* = .16	*r* = -.18, *p* = .17
Experiment 5 (PARH)	48^†^	17.28 ± 9.58	4.18–47.13	1.56	2.93	*r* = -.17, *p* = .26	*r* = -.21, *p* = .15
Experiment 6 (Verbal Check)	62	26.30 ± 9.27	13.75–42.80	1.39	2.23	*r* = -.14, *p* = .29	*r* = .29, *p* = .02*
Experiment 7 (category)	33	22.61 ± 9.92	3.68–53.22	1.44	3.11	*r* = -.12, *p* = .49	*r* = -.06, *p* = .75
Experiment 7 (initial-letter)	34	21.97 ± 9.34	2.55–40.27	-0.46	0.12	*r* = -.35, *p* = .04*	*r* = -.01, *p* = .97
Experiment 7 (syllable)	32	23.77 ± 7.35	3.31–36.73	-0.92	1.63	*r* = .38, *p* = .04*	*r* = -.09, *p* = .61
Experiment 8 (RM control)	30	25.14 ± 10.79	8.55–51.57	0.82	0.25	n/a	n/a
Experiment 8 (PM standard)	30	30.78 ± 13.77	17.54–83.82	2.33	6.92	n/a	n/a
Experiment 8 (PM-Encoding-Probe)	89	24.24 ± 7.80	13.64–54.72	1.45	2.69	*r* < .01, *p* = .98	*r* = -.07, *p* = .52

Abbreviations: PARH: Perceived Awareness of the Research Hypothesis scale; PM = prospective memory; SD = standard deviation; RM: retrospective memory

^†^ indicates exclusion of extreme outliers (>100 sec encoding or <3 sec encoding).

* indicates correlational *p* < .05

### Discussion

One view of prospective memory encoding emphasizes strategic/elaborative processes; however, at least 20% of participants reported that they hardly thought about the task at all. An even higher percentage of participants showed “off task” thoughts (mind wandering) immediately following the prospective memory instructions, even though the key to advance from the encoding screen to the thought probe screen was Q (i.e., the prospective memory response key). Therefore, in laboratory experiments, the encoding of prospective memories is conscious, but very short lived (transient). Interestingly, the participants who generated specific exemplars into their intention plan did not require additional time to do so (i.e., encoding duration), again indicating that some components of prospective memory encoding can be quick and cursory (perfunctory).

## Experiment 4

We next tested for age effects on intention encoding processes. If encoding is strategic/elaborative, or otherwise cognitively-demanding, then older adults should show more frequent mind wandering [51] and generate fewer specific exemplars (as in category fluency tests; 40]. Alternatively, if intention encoding can be perfunctory, then there should be no age differences in intention encoding [18].

### Method

We recruited 128 adults who were living in the United States via Amazon’s Mechanical Turk (MTurk). Studies that compared data collection in the laboratory versus MTurk supported the validity of internet-based data collection [52]. Multiple prospective memory studies have been performed online [53,54]. Nevertheless, we restricted participation to MTurk workers with a 95%-100% approval rating, which increases data quality [55]. We excluded 13 participants whose ages diverged from the range specified during study advertisement for young adults (ages 18–30, 25.86 ± 2.65) and older adults (ages ≥60, 64.50 ± 4.97). Though the older adults reported to taking significantly more prescription medications (*M*_Older_ = 1.78 ± 2.13) than the young adults (*M*_Younger_ = 0.30 ± 1.06), *t*(86.48) = 4.74, *p* < .001 (corrected for unequal variances), the age groups were similar in years of education (*M*_Younger_ = 14.69 ± 2.18, *M*_Older_ = 15.02 ± 2.98, *t* < 1), percentage of female participants (Younger: 32.73%; Older: 43.33%, χ^2^(1) = 1.37, *p* = .24), percentage of non-white participants (Younger: 23.64%; Older: 18.33%, χ^2^ < 1), ratings of their health on a 1–5 scale (*M*_Younger_ = 3.60 ± 1.06, *M*_Older_ = 3.57 ± 1.03, *t* < 1), reported number of hours slept the previous night (*M*_Younger_ = 7.27 ± 1.00, *M*_Older_ = 7.03 ± 1.09, *t*(113) = 1.22, *p* = .23), and reported exercise frequency on a 1–4 scale (*M*_Younger_ = 2.73 ± 0.80, *M*_Older_ = 2.45 ± 0.83, *t*(113) = 1.81, *p* = .07). Therefore, the older adults in the current study were generally very healthy.

All procedures mirrored Experiment 3’s no-prime condition except that participants completed questionnaires after the encoding thought probe procedure. The statistical analyses mirrored Experiments 1–3, with the addition of Bayesian analyses to statistically support the null hypothesis of no age effects. BF_10_ < 1 is evidence in favor of the null hypothesis (i.e., no age differences in encoding) whereas BF_10_ > 3 is substantial evidence for the alternative hypothesis (i.e., age differences in encoding). We conducted Bayesian analyses using JASP software [56].

### Results

As shown in Tables 2 and 3, there were no significant differences between young and healthy older adults in specific exemplar generation (BF_10_ = 0.32), off-task mind wandering (BF_10_ = 0.42; less mind wandering overall in this MTurk sample), or any other aspect of prospective memory encoding (all χ^2^s < 2, *p*s > .10). The healthy older adult group (1.05 ± 2.08) generated nominally, but not significantly, more specific exemplars than the young adult group (0.62 ± 1.15; *t*(113) = 1.36, *p* = .18, *d* = .26, BF_10_ = 0.46). Evidence in favor of the null was particularly strong when, based on the semantic fluency literature [40], the tested hypothesis was set to young adults being expected to generate more exemplars, BF_10_ = 0.09. Table 4 shows that there were no significant associations between encoding duration and likelihood of generating specific exemplars in young or healthy older adults (see Fig 3 for encoding data across experiments). Therefore, the results of Experiment 4 suggested that prospective memory encoding need not always be cognitively demanding, but may instead be perfunctory/transient.

## Experiment 5

One potential concern is that task demand characteristics cause participants to later say that they generated specific fruit words. For example, if participants believe the research hypothesis to be about specific exemplar encoding, then that would bias the results rather than indicate that some components of encoding can be perfunctory/transient. To investigate this demand-characteristic-view, we administered an established quantitative measure of demand characteristics [57] following the encoding thought probe procedure.

### Method

Adult participants (N = 59, ages 26.56 ± 3.61) living in the United States were recruited via MTurk according to the specifications described in Experiment 4. The procedure was identical to Experiment 4, with the addition of the Perceived Awareness of the Research Hypothesis scale (PARH [57]). The PARH requires participants to rate four statements on a 7-point scale (1 = Strongly Disagree, 7 = Strongly Agree), such as “I had a good idea about what the hypotheses were in this research.” If the mean score is below 4, then that indicates that participants were unclear about the hypotheses and that demand characteristics do not explain the study findings [57]. Following the rating scale, we also asked participants to free respond to the question “What do you think the researchers were trying to demonstrate with this study?”

### Results

In the free responses, a few participants showed partial knowledge of the hypotheses on encoding (e.g., “I honestly have no idea. Maybe trying to see if I thought of fruits as a general topic or more specifically? I really have no idea”). However, the most common response (23 of 55 provided responses) was a variant of “I honestly have no idea.” Importantly, PARH scores (2.70 ± 1.57) were significantly below the cutoff value of 4.0, *t*(58) = 6.34, *p* < .001, *d* = 1.66, indicating minimal demand characteristics. Individuals who reported generating specific exemplars (3.05 ± 1.15) showed similar PARH scores as individuals who did not (2.48 ± 1.77; *t*(56.97) = 1.52, *p* = .14, *d* = .39, Yates’ correction). There were outlier data points for encoding duration (<3 or >100 seconds), but regardless of whether these data points were excluded, encoding duration did not significantly differ across specific exemplar generators or non-generators (see Table 4 and Fig 3). Furthermore, there was no association between encoding duration and specific exemplar generation when only examining participants who were not mind wandering (*r*(22) = -.14, *p* = .51). Thus, demand characteristics do not explain participants’ perfunctory/transient encoding of prospective memory intentions.

## Experiment 6

In all preceding experiments we have assumed that the prospective memory intention was consciously encoded prior to assessing perfunctory/transient processes (cf. [18]). In Experiment 6, we experimentally confirmed conscious encoding by having participants verbally explain the prospective memory instructions to the experimenter. The idea here is that the verbal experimenter-check provides a strong test of the robustness of perfunctory/transient processes.

### Method

Sixty-two Baylor University undergraduate students participated in a cognitive laboratory setting. The procedure was identical to the no-prime condition in Experiments 3–5 except that participants were required to verbally explain the prospective memory task to the research assistant prior to completing the thought probe questions. Verbal explanation was not considered complete until participants had spoken the prospective memory cue (fruits) and response key (Q). Afterward, the experimenter advanced the screen so that participants could respond to the thought probe questions. Research assistants were masked to the study’s hypotheses.

### Results

Despite requiring participants to verbalize their general intention, Table 2 shows that mind wandering reports remained prevalent, demonstrating the transient nature of encoding processes. Furthermore, even though participants spent longer encoding their intention, including speaking their intention to the experimenter, specific exemplar generation occurred at similar rates as previous experiments (and was unrelated to encoding duration, even when off-task participants were excluded, *r*(27) = -.17, *p* = .39; see also Table 4 and Fig 3). These findings converge with the notion that specific exemplar encoding is more perfunctory than strategic.

## Experiment 7

Better understanding of encoding processes will inform theoretical and methodological issues within the prospective memory field. According to the Multiprocess Framework [58,59], the overlap between how a target cue is encoded and how it is processed at retrieval determines the extent to which one must rely on strategic monitoring versus spontaneous retrieval processes (*cue focality hypothesis* [60]). A typical example of a *focal* cue would be the target word “horse” during a task that requires processing of whole words (lexical decision task) whereas an example of a *nonfocal* cue would be detecting words that begin with the letter “h” during a lexical decision task. Fruit and animal category cues have nearly always been classified as *nonfocal* to ongoing tasks in review papers [61] and in meta-analysis articles [62]. However, in Experiments 1–6, many participants reported generating specific exemplars, which could transform a categorical intention from being a nonfocal cue into a focal cue. Therefore, it is pertinent to prospective memory theories to assess whether other cue types typically classified as “nonfocal” (i.e., during a lexical decision task) elicit similar variability in encoding processes.

In Experiment 7, we compared encoding processes for categorical cues relative to syllable cues and initial-letter cues. One hypothesis is that any cue type should encourage participants to generate specific exemplars (except for “exact” cue types, such as the specific cue word “table”), particularly if affirmative responses are due to task demand characteristics. An alternative hypothesis is that the superordinate, semantic (fruit) category triggers spreading activation to specific exemplars, and thus, participants may be less likely to generate specific exemplars of syllable and initial-letter cues in a perfunctory manner.

### Method

Ninety-nine Baylor University undergraduate students were randomly assigned to the fruits category, the syllable cue, and the initial-letter cue conditions. The practice block did not contain any prime words, prime letters, or prime syllables. The category cue procedure was identical to that used in the no-prime condition in Experiment 3 (Fig 1). The instructions for the initial-letter condition were as follows (syllable cue condition in brackets):

In this experiment, we are also interested in your ability to remember to perform an action at a given point in the future. Therefore, during the word/nonword task, we would like you to perform a special action whenever you see an item that BEGINS with the letter T [item that includes the syllable "tor"]. Whenever you see an item that begins with the letter T [includes the syllable tor], you should remember to press the 'Q' key. Press 'Q' to continue.

#### Statistical analysis

For free response and forced-choice response data, we conducted planned comparisons between the categorical cue, initial-letter cue, and syllable cue conditions individually. For the encoding duration data, we conducted a series of between-subjects analyses of variance (ANOVAs) to evaluate whether condition and/or encoding type (specific) related to encoding duration.

### Results

#### On-mind free responses

As shown in Table 2, mind wandering (off-task responses) did not significantly differ across conditions (all χ^2^ < 1.3, *p*s > .10).

#### Specific exemplar generation

Specific exemplar generation occurred in the categorical cue condition, χ^2^(1) = 10.91, *p* < .001 (Yates’ correction), and the syllable cue condition, χ^2^(1) = 7.00, *p* = .008 (Yates’ correction), but not significantly in the initial-letter cue condition, χ^2^(1) = 2.39, *p* = .12 (Yates’ correction; Table 3). The direct comparison between proportion of specific exemplar generators in the categorical cue and initial-letter cue conditions was less definitive, χ^2^(1) = 3.33, *p* = 0.07 (Yates’ correction). However, when measuring the total number of fruits generated, a large reduction was clearly evident from the categorical cue condition (1.06 ± 1.71) to the initial-letter condition (0.18 ± 0.72), *t*(42.60) = 2.74, *p* = .009, *d* = 0.84 (corrected for unequal variances). The mean number of specific exemplars generated did not differ between the syllable cue condition (0.59 ± 1.41) and the other two conditions (*p*s > .10). The initial-letter cue participants were overall less likely to respond affirmative than the categorical cue participants for the general category question, χ^2^(1) = 5.81, *p* = .02, but importantly, when forced to choose whether they focused more on generating specific exemplars or on the overarching category, participants in the initial-letter cue condition were less likely to be biased toward specific exemplar generation than those in the categorical cue condition, χ^2^(1) = 4.30, *p* = .04 (Yates’ correction; no significant differences relative to the syllable condition, *p*s > .10).

Some readers may be surprised that specific exemplar generation was not also reduced in the syllable cue condition. We identified a counterbalance effect in the syllable cue condition regarding whether participants were first asked if they generated specific exemplars or first asked if they thought of cues as a general category (no counterbalance effects in the initial-letter condition, *p*s > .10). When the specific exemplar question was asked first, there was not a statistical difference in specific exemplar generation between the syllable cue (50.0%) and categorical cue (33.3%) conditions (χ^2^ < 1). When the general category question was asked first, on the following screen, none of the syllable cue participants stated that they generated specific exemplars. This 0% of syllable cue participants was significantly lower than the 33.3% of categorical cue participants who were in the same counterbalance order, χ^2^(1) = 4.13, *p* = .04. These counterbalance patterns might be spurious (Type I error), they might reflect differential difficulty understanding the questions asked, or they might simply indicate that syllable cues are less likely to trigger specific exemplar generation under some conditions.

#### Encoding duration

Mean encoding duration was similar across the three cue conditions (all *t*s < 1; Table 4), implying that the group differences in specific exemplar generation were not explained simply by alterations in strategic/elaborative encoding processes. Interestingly, there was a significant interaction between cue condition and whether participants indicated that they generated specific exemplars, *F*(2, 93) = 4.07, *MSE* = 76.03, *p* = .02, η_p_^2^ = .08 (the main effect of specific exemplar generation was not significant, *F*<1). In the *categorical* condition, specific exemplar generation was unrelated to encoding duration, as in the previous experiments (Fig 3; *t* < 1; Specific-Yes = 20.90 ± 5.36; Specific-No = 23.46 ± 11.57). For the *syllable* cue condition, participants who spent longer encoding the prospective memory instructions were significantly more likely to generate specific exemplars, *t*(30) = 2.21, *p* = .03, *d* = 0.81 (Specific-Yes = 28.46 ± 5.04; Specific-No = 22.20 ± 7.41). The reverse pattern was observed in the *initial-letter* condition, but there were only four exemplar-generators in this condition *t*(32) = 2.11, *p* = .04, *d* = 0.75 (Specific-Yes = 13.16 ± 11.79; Specific-No = 23.14 ± 8.54). These data suggest that whether exemplar generation is strategic/elaborative versus perfunctory/transient depends on the prospective memory cue type.

### Discussion

Some intentions may be more easily formed “in passing” than others. Relative to categorical cues, other initial-letter and syllable cue conditions elicited fewer specific exemplars. This experimental effect converges with Experiment 5 in showing that demand characteristics do not lead participants to respond affirmative to the specific exemplar generation question. Interestingly, the relationship between encoding duration and specific exemplar generation differed across cue types: Exemplars of category cues may be encoded in a perfunctory manner whereas exemplars of syllable cues require strategic/elaborative processing. The theoretical implication is that encoding processes not only vary across individuals, but also across different cue types, even for cue types that have historically been classified together as *nonfocal*.

## Experiment 8

A remaining question is whether encoding processes predict later retrieval. Prospective memory researchers distinguish between top-down monitoring processes, and bottom-up spontaneous retrieval processes [59]. For example, one might effortfully maintain a prospective memory intention in working memory (pick up groceries) and monitor for potential retrieval cues (grocery store signs). Because monitoring is a controlled process that requires working memory resources that would normally be devoted to ongoing activities (e.g., driving), monitoring incurs a *cost* to ongoing task performance (e.g., slowed response times [63]).

Monitoring is a cognitively demanding process, and therefore, individuals tend not to monitor continuously across long retention intervals [64–68]. In the absence of monitoring, prospective memories can still sometimes be spontaneously retrieved. For example, we [69] instructed participants to remember to press the Q key if they ever saw the word crossbar (focal condition) or a word beginning with the letter c (nonfocal condition), and then had them perform 500 lexical decision trials before presenting crossbar. Monitoring costs were absent by trial 501, yet approximately ¾ of participants in the focal condition still remembered to press the *Q* key, relative to fewer than ¼ in the nonfocal condition (see also [70]). Thus, cue focality is considered a discriminating factor between whether an individual can successfully rely on spontaneous retrieval versus needing to monitor for cues.

In Experiment 8, after the thought probe procedure, participants performed a 500-trial lexical decision block, with the first target event on trial 501. We predicted that specific exemplar generators would outperform non-generators (Hypothesis 1) because categorical cue studies have observed greater prospective memory performance when highly-typical versus atypical categorical cues were presented [37,39,43,71,72].

We also included a retrospective-memory comparison group that did not encode the prospective memory task. This comparison group allowed us to determine monitoring costs for the prospective memory group [69]. The cue focality hypothesis would predict monitoring cost to be present in individuals focused on fruits as a general category, but reduced or absent in individuals focused on specific examples of fruits (Hypothesis 2).

Several design challenges emerge with directly connecting thought-probe encoding processes to later performance (cf. [73]). For example, participants might generate specific exemplars that are not later presented, and doing so would be expected to trigger retrieval-induced forgetting [74]. We avoided this pitfall by selecting 10 highly-typical exemplars of fruits to be successively presented (beginning on trial 501). Another challenge is that the encoding thought probes might change how participants approached the task, for example, by instilling more importance to the prospective memory task (for discussion, see Kliegel et al.’s plan aloud procedure [75]). To address the general issue of the thought probes increasing task importance, we included a “standard” prospective memory comparison condition in which encoding processes were not assessed, but all other procedural elements were maintained. If the encoding thought probes increased strategic processing (cf. importance effects [76]), then the group with encoding thought probes should outperform the standard prospective memory condition.

### Method

#### Participants

Baylor University undergraduate students (*N* = 149) participated for partial class credit. Participants were randomly assigned to the following conditions: retrospective memory control (*n* = 30), standard prospective memory (*n* = 30), and PM-Encoding-Probes (*n* = 89). A larger sample size was recruited for the PM-Encoding-Probes condition to ensure reasonable subgroup sizes (i.e., given the frequencies in Table 3, we expected a minimum of *n* = 20 to generate specific exemplars).

#### Materials

Lexical decision task filler items were the same as used in a previous study [69]. Highly typical fruit prospective memory words were selected using semantic norm databases [47,77].

#### Procedure

After being introduced to the lexical decision task and performing a practice block, participants completed a pre-encoding, control block of 100 lexical decision trials. No fruit prime words appeared during practice or baseline/control blocks.

Participants in the prospective memory conditions were next instructed that they would perform another lexical decision block, but to remember to press the *Q* key if they ever see any fruit words. Participants in the *retrospective*-memory control condition were instructed:

“In this experiment, we are also interested in your ability to remember certain "target" keys and categories. Your target key is "Q" and your target category is "fruits." At the end of the experiment, we will ask you to recall your target key and target category. Press 'Q' to continue.”

In the PM-Encoding-Probes condition, we then presented the free response and yes/no questions shown in Fig 1. All participants then completed 510 lexical decision task trials. Apple was presented on trial 501, followed by the following fruit words: cherry, orange, peach, banana, berry, pear, plum, kiwi, and apple (Experiment 3 showed apple to be the most commonly generated fruit exemplar, and so we presented it twice to maximize the probability of a retrieval). We selected the procedure of having all targets at the end of the block rather than early to minimize strategic monitoring processes; if a target cue is presented early it will trigger more monitoring, and perhaps additional attempts at cue generation for the remainder of the block [68]. Though participants were allowed to press the Q key immediately upon seeing the target, or after making their ongoing task response, pressing the Q key advanced the screen, so functionally, participants could make the Q response instead of an ongoing task response. After the prospective memory experiment, a subset of participants (*n* = 116) completed the automated reading span task to estimate working memory capacity [78].

#### Statistical analyses

For prospective memory performance, we calculated the proportion of fruit target trials in which the *Q* key was pressed. For ongoing task cost, we used the same analysis of covariance (ANCOVA) approach as in our previous study [69]: We calculated mean response times to all trials with correct responses and covaried response times from the pre-encoding, control block. To complement the “untrimmed” response time analyses, we also trimmed response times ±2 standard deviations from each individual’s (sub)block mean, because trimmed response times are sometimes considered to be more sensitive to group differences (lower variance). Wherever trimmed response times led to a different statistical conclusion (alpha = .05) than untrimmed response times, we present those data. We planned to compare prospective memory performance and ongoing task cost as a function of encoding subgroups (yes/no, encoding bias questions), and further planned to compare these subgroups against the retrospective-memory control group. Because we identified *pre*-*experimental* group differences in the standard prospective memory condition relative to the other conditions, we report those data separately.

### Results

#### Encoding thought probe responses

The thought probe data converged with Experiments 1–7 and are shown in Tables 2 and 3 (Fig 2 shows the data collapsed across experiments). Though theories of planning emphasize the role of working memory capacity [79], reading span scores were not associated with specific exemplar generation (Specific-Yes: 56.64 ± 9.80, Specific-No: 55.35 ± 11.32, *t* < 1, BF_10_ = 0.30) or encoding the fruit cue as a general category question (General-Yes: 56.98 ± 9.65, General-No: 52.75 ± 13.00, *t*(57) = 1.36, *p* = .18, *d* = .36, BF_10_ = 0.61). Moreover, reading span scores did not significantly distinguish on-task participants (57.28 ± 9.12) from participants who were mind wandering (52.79 ± 13.23; *t*(26.42) = 1.34, *p* = .19, *d* = .52, BF_10_ = 0.72, corrected for unequal variances). These data converge with the view that prospective memory encoding can be perfunctory.

#### Frequency of cue words generated

Nearly all the specific-exemplar-generator participants (93.9%) encoded a fruit word that would be a prospective memory target word. The most frequently generated fruits were banana, apple, and orange. Of participants who generated specific fruits, participants listed 2.45 ± 1.28 fruit words.

#### Encoding duration

According to the strategic/elaborative view, because prospective memory is *future* oriented, it may prompt greater imaginal-enactive processes at encoding than retrospective memory encoding [80]. However, as shown in Table 4, encoding duration did not significantly differ across the PM-Encoding-Probes and retrospective memory control conditions (*t* < 1; cf. [81]). There were also no associations between encoding duration and encoding thought probe responses (all *p*s > .10; see Fig 3). Furthermore, if successful intention encoding requires strategic/elaborative processing, then longer encoding durations should predict better prospective memory performance; however, encoding duration correlated negatively (nonsignificantly) with later performance (*r*_*p*_(116) = -.14, *p* = .14, controlling for condition). Thus, forming a category-cue intention does not require more strategic processing than reading a similar length instruction screen, and even perfunctory encoders can be successful prospective memory performers.

#### Standard condition showed pre-experimental differences

Despite random assignment to conditions, and identical instructions, the standard condition took significantly longer to encode the prospective memory task than the PM-Encoding-Probes condition, *t*(35.47) = 2.47, *p* = .02, *d* = .83 (corrected for unequal variances). Moreover, during the control lexical decision block (Tables 5 and 6), the standard condition showed slower response times than the retrospective-memory condition, *t*(42.01) = 2.59, *p* = .01, *d* = 0.79 (corrected for unequal variances) and PM-Encoding-Probes condition, *t*(117) = 1.85, *p* = .07, *d* = 0.34. For prospective memory responses, in the standard condition, 90% of participants remembered to press *Q* at least once and there were significantly more overall *Q* responses to fruit words (*M* = .73) than in the PM-Encoding-Probes condition, *t*(66.21) = 3.24, *p* = .002, *d* = 0.79 (corrected for unequal variances). It is unclear why this condition was so aberrant, but the direction of the results was opposite of the prediction that the thought probe questions would increase the importance of the prospective memory task.

#### Prospective memory performance relative to encoding processes

**Fig 4:**
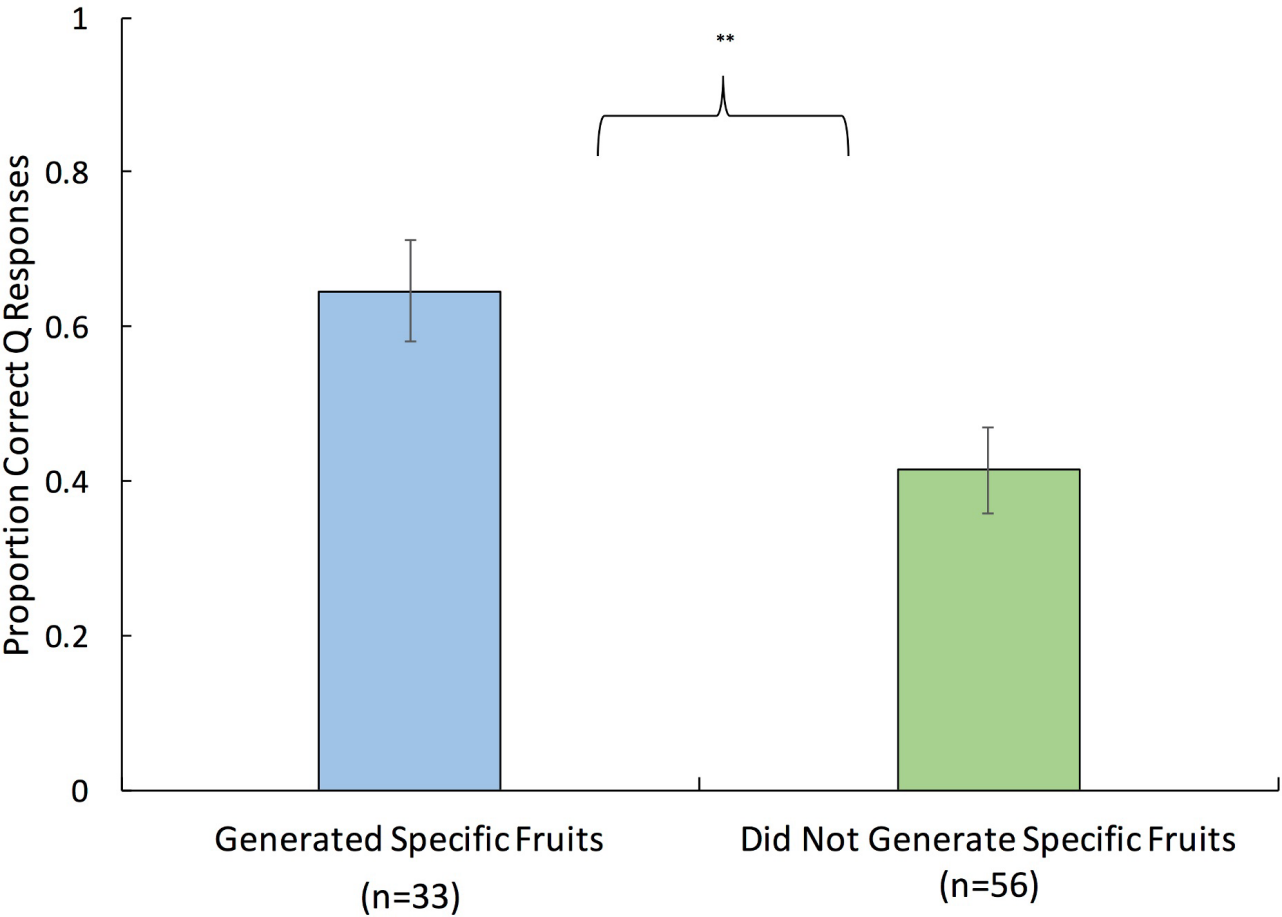
Prospective memory performance in Experiment 8 as a function of specific exemplar encoding. Error bars reflect standard errors and ** indicates *p* < .01.

In the PM-Encoding-Probes condition, one hypothesis was that specific exemplar generation would increase prospective memory performance. As illustrated in Fig 4, participants who reported generating specific exemplars performed significantly better than those who did not, *t*(72.41) = 2.68, *p* = .009, *d* = 0.63 (corrected for unequal variances). Moreover, participants who generated specific exemplars and indicated that they were biased toward specific encoding (0.69 ± 0.35) significantly outperformed those who did not generate specific exemplars and reported being biased toward categorical processing (0.41 ± 0.43), *t*(36.85) = 2.41, *p* = .02, *d* = 0.79 (corrected for unequal variances).

If successful encoding always requires the engagement of strategic/elaborative processes, then participants who reported that they hardly thought about the prospective memory task (at encoding) should perform very poorly. By contrast, performance did not differ as a function of responses to the encoding bias question (Hardly Thought About It = 0.53 ± 0.40; Exemplar Bias = 0.54 ± 0.42; Category Bias = 0.46 ± 0.43; *p*s > .10).

#### Ongoing task performance

A second hypothesis was that encoding biases might alter subsequent retrieval processes (monitoring versus spontaneous retrieval), as measured by ongoing task performance. Typically, ongoing task accuracy is not a sensitive measure of monitoring, and Table 5 shows that accuracy cost did not significantly differ across the PM-Encoding-Probes condition and the retrospective-memory control condition (*F* < 1) or as a function of encoding thought probe responses (largest *F*(1, 63) = 2.17, *MSE* = .006, *p* = .15, η_p_^2^ = .03, for encoding bias question).

**Table 5:** Ongoing task accuracy in Experiment 8 (proportion correct means ± standard deviations).

Condition	Control block	PM block 1–100	PM block 101–200	PM block 201–300	PM block 301–400	PM block 401–500	PM block Overall
Retrospective memory (n = 30)	.86±.08	.81 ± .11	.80 ± .09	.84 ± .10	.84 ± .09	.81 ± .11	.82 ± .09
PM Standard (n = 30)	.86±.08	.82 ± .10	.80 ± .08	.84 ± .10	.82 ± .10	.81 ± .10	.82 ± .09
PM-Encoding-Probes (n = 89)	.87±.06	.85 ± .08	.82 ± .09	.84 ± .10	.82 ± .10	.81 ± .10	.83 ± .08
Specific Yes (n = 33)	.88±.06	.86 ± .07	.84 ± .07	.85 ± .08	.83 ± .07	.83 ± .08	.84 ± .07
Specific No (n = 56)	.87±.07	.84 ± .08	.82 ± .09	.83 ± .11	.81 ± .11	.80 ± .11	.82 ± .09
General Yes (n = 68)	.88±.06	.85 ± .08	.83 ± .08	.85 ± .08	.83 ± .08	.82 ± .09	.84 ± .08
General No (n = 21)	.86±.07	.84 ± .09	.81 ± .10	.81 ± .13	.79 ± .14	.78 ± .12	.81 ± .11
General Bias (n = 43)	.87±.07	.85 ± .08	.83 ± .07	.85 ± .09	.84 ± .08	.83 ± .09	.84 ± .07
Specific Bias (n = 22)	.86±.07	.83 ± .10	.80 ± .12	.82 ± .11	.79 ± .12	.80 ± .12	.81 ± .11
Didn’t think about PM (n = 24)	.88±.05	.86 ± .05	.83 ± .06	.82 ± .10	.81 ± .11	.80 ± .10	.83 ± .07

Abbreviations: PM = prospective memory

**Fig 5:**
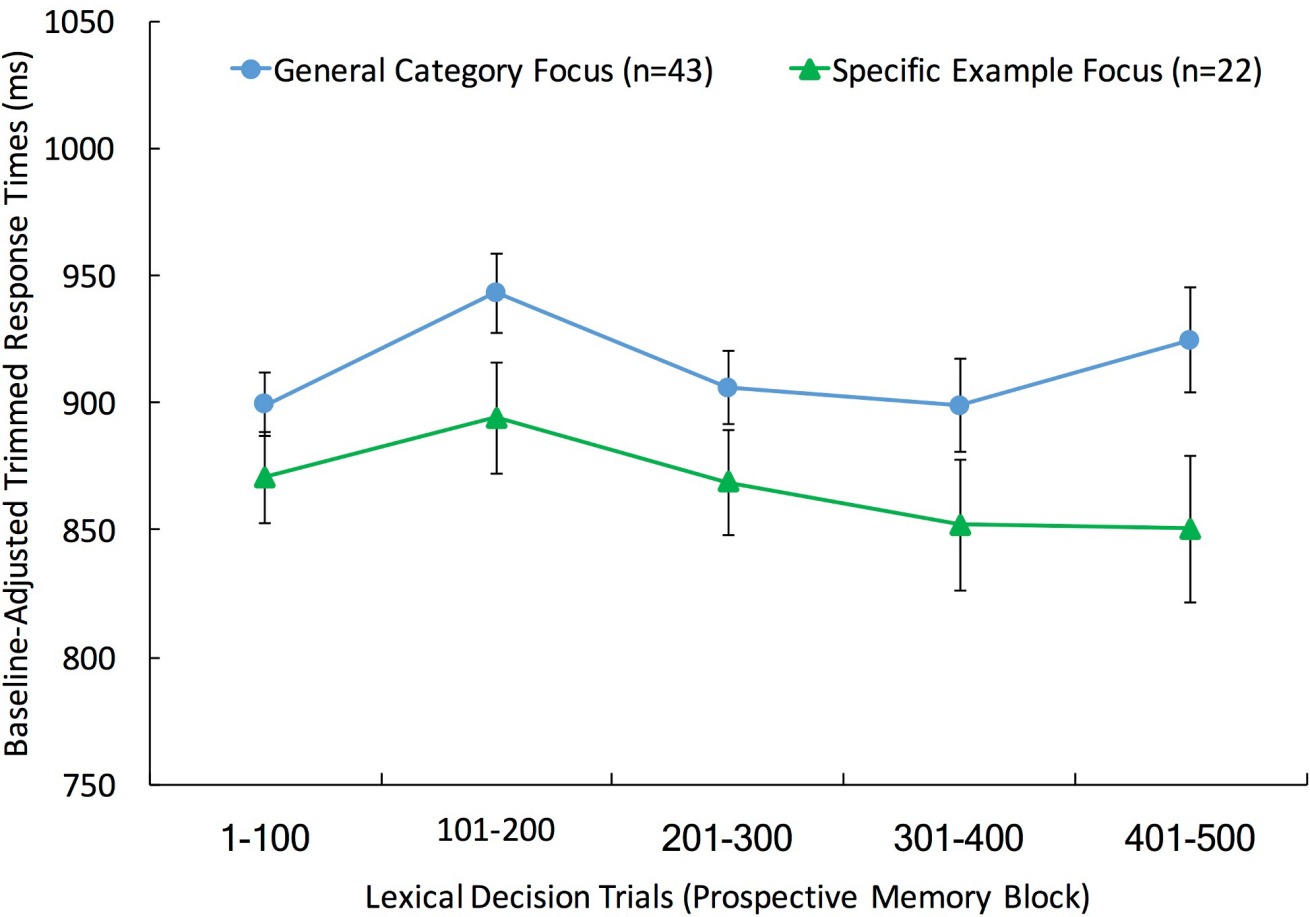
Ongoing task cost as a function of encoding processes. Baseline-adjusted mean trimmed responses times across quintiles of the prospective memory test block in Experiment 8. The cost results are separated by individuals focused on fruits as a general category and individuals focused on specific fruit exemplars. Error bars represent standard errors.

Table 6 presents the unadjusted and untrimmed mean response times on correct, non-target lexical decision trials. Response time cost did not differ across the PM-Encoding-Probes condition and the retrospective-memory control condition, or as a function of individuals’ responses to the specific exemplar and general category questions (all *F*s < 1). However, as illustrated in Fig 5, separating participants based on the encoding bias question demonstrated that participants who focused on fruits as a general category tended to show greater cost than those who focused on specific fruit exemplars (trimmed response times: *F*(1, 62) = 4.02, *MSE* = 8393.10, *p* < .05, η_p_^2^ = .06; untrimmed: *F*(1, 62) = 3.73, *MSE* = 11538.33, *p* = .06, η_p_^2^ = .06). Furthermore, there was evidence for a greater group difference in response time cost late in the prospective memory block (trials 401–500; trimmed response times: *F*(1, 62) = 4.36, *MSE* = 18070.19, *p* = .04, η_p_^2^ = .07; untrimmed: *F*(1, 62) = 3.52, *MSE* = 22459.00, *p* = .07, η_p_^2^ = .05) relative to early in the prospective memory block (trials 1–100; *F*(1, 62) = 1.69, *MSE* = 6970.64, *p* = .20, η_p_^2^ = .03; untrimmed: *F*(1, 62) = 2.36, *MSE* = 9105.61, *p* = .13, η_p_^2^ = .04), though the direct test for the block by group interaction was nonsignificant (*F*(1, 62) = 1.08, *MSE* = 8394.04, *p* = .30, η_p_^2^ = .02).

**Table 6:** Response times on correct, non-target ongoing task trials in Experiment 8 (means ± standard deviations).

Condition	Control block	PM block 1–100	PM block 101–200	PM block 201–300	PM block 301–400	PM block 401–500	PM block Overall
Retrospective memory (n = 30)	881±102	908±113	954±123	943±98	923±120	945±123	935±103
PM Standard (n = 30)	991±210	1071±229	1053±212	1024±211	995±186	993±152	1029±184
PM-Encoding-Probe (n = 89)	913±197	941±190	990±218	961±217	945±224	958±206	961±197
Specific Yes (n = 33)	925±208	934±161	1002±213	968±209	963±232	969±168	968±189
Specific No (n = 56)	906±191	945±207	983±223	957±223	934±220	952±226	957±204
General Yes (n = 68)	927±216	947±207	1004±237	974±222	956±229	973±207	972±211
General No (n = 21)	868±105	921±124	946±137	921±196	907±204	908±198	925±146
General Bias (n = 43)	903±156	941±191	999±217	960±178	954±199	978±206	968±188
Specific Bias (n = 22)	962±269	952±194	995±231	971±241	935±245	938±153	960±202
Didn’t think about PM (n = 24)	888±185	931±194	970±216	955±262	936±253	940±249	950±217

Abbreviations: PM = prospective memory

### Discussion

Inter-individual variability in encoding was associated with prospective memory performance (Hypothesis 1) and retrieval processes (Hypothesis 2). Consistent with the Multiprocess Framework, participants who generated specific exemplars at encoding (focal cues) showed significantly greater prospective memory performance than those who did not [37,39,43,71,72]. However, because the specific exemplar feature was quasi-experimental (cf. [75]), we cannot rule out that “participants who show good prospective memory are also good planners” (p. 1737 [75]). For example, perhaps participants who generated specific exemplars were more motivated to perform the prospective memory task. If so, then based on previous work [76], specific-exemplar encoders should have shown more ongoing task costs, higher working memory scores, or altered encoding durations. By contrast, individuals who focused on specific fruit cues (focal cue) demonstrated fewer monitoring costs than those that focused on fruits as a general category (nonfocal cue), with no group differences in encoding duration or working memory scores. Relative to the retrospective-memory control condition, specific-exemplar encoders showed no ongoing task costs, indicating that spontaneous retrieval processes supported their prospective remembering [58]. Though additional research is warranted, the collective findings are more consistent with the cue focality account than a motivation account.

Consistent with the perfunctory/transient view, there was minimal-to-no evidence that prospective memory performance suffered in participants who were mind wandering, who had low working memory capacity, or who reported to hardly thinking about the prospective memory task. These results distinguish prospective memory encoding from theoretical views in the planning literature [79] and the retrospective memory encoding literature [82–85]. Even the literature on goal fulfillment, which argues that many individuals form general intentions (with minimal cognitive effort), predicts that strategic/elaborative processes are beneficial, if not necessary, for later goal execution [86]. Prior to conducting the current work, we would have assumed that categorical prospective memory encoding constitutes “deep” processing [19], but the totality of findings on mind wandering, brief encoding durations, and null associations between mind wandering and prospective memory performance converge on the conclusion that at least some components of intention encoding can be perfunctory/transient.

## Conclusions

We investigated the encoding of prospective memory intentions using a thought probe procedure that has previously been useful in examining retrieval processes [33–35]. As a theoretical orientation, we contrasted two general views. The elaborative/strategic view, which emanates from the literature on planning and retrospective memory and emphasizes the functional importance of effortful, working memory resources. By contrast, the perfunctory/transient view emphasizes that some components of prospective memory intentions might be encoded with minimal effort. The consistent theme across eight experiments was that there exists substantial quantitative and qualitative variability in the manner in which participants encode laboratory prospective memory intentions. Whereas quantitative differences in encoding duration seemed to have minimal functional value, differences in encoding quality clearly mattered: Intentions that were encoded more specifically were more likely to be later remembered with lower or no cost (Experiment 8). In other words, the most effective form of encoding occurred in a perfunctory manner.

### Transience of prospective memory encoding

Task disengagement, or mind wandering, is common in classrooms and during psychology experiments [87,88]. It is surprising, however, that over 40% of free responses were solely off-task (Fig 2). Our procedure was not a long, monotonous task, as is the case in many mind wandering studies. Furthermore, the prospective memory instructions are arguably the most important stage of a prospective memory experiment. Obviously, this stage is more important to scientists than to most participants. A potential caveat is that some participants who were classified as “off-task” may have initially been engaged. But, it seems highly unlikely that all of the participants categorized as off-task were engaging strategic/elaborative encoding processes: Nearly one-quarter of participants reported that they hardly thought about the prospective memory task at all (Fig 2).

Similar levels of hardly-thinking-about-encoding have been reported in naturalistic studies. For example, in a naturalistic study of eight participants, Holbrook and Dismukes [89] found that for 23% of intentions that participants “did not think very much about the intention, just assumed [they] would remember to perform it” (see also, Marsh and colleagues’ [31] study of “recorders” and “nonrecorders”). Such participants performed poorly in their study [89], but in other naturalistic research, participants who only implicitly formed an intention to put their watch back on their wrist were able to successfully remember that intention [18].

### Categorical cues: Focal, nonfocal, neither, or both?

Even when participants were “on-task,” they differed in how they encoded the prospective memory cue. Some researchers have acknowledged that participants might generate specific exemplars during category prospective memory encoding [39,43,90], but many scientific reports that used categorical cues have dismissed or otherwise ignored this possibility. Our review papers and others’ meta-analysis papers have always classified categorical cues as “nonfocal” to ongoing tasks [61,62]. Therefore, a salient finding from the encoding thought probe procedure was the robustness of specific exemplar generation in all experiments (Fig 2). Particularly relevant to prospective memory’s cue focality hypothesis [60], in Experiment 8, we observed that the variability in encoding specificity mattered to prospective memory accuracy and ongoing task cost: The more specifically a categorical cue was encoded, the more likely it was to elicit performance akin to a focal-cue condition. Thus, encoding variability may explain why categorical cues can sometimes trigger spontaneous retrieval [91] and be associated with minimal age differences in prospective memory performance [92]. Indeed, in Experiment 4, we found that healthy older adults were as likely as young adults to encode specific exemplars.

The methodological implication for future research on cue focality may be to use initial-letter cues. Perceptual identification studies indicated that initial-letters were as easily identifiable as whole words, which are the prototypical focal cue [69]. In addition, in Experiment 7, specific exemplar generation was reduced with initial-letter cues relative to categorical cues, possibly because superordinate categories (animals, fruits) cause spreading activation in semantic networks to a category’s exemplars [49,50]. To be clear, we are not arguing that researchers should never use categorical cues. Instead, we recommend using categorical cues to investigate encoding variability, encoding—retrieval interactions, and similar questions (but not to investigate cue focality).

### Strategic versus perfunctory: Dichotomy or continuum?

In the current work, we described strategic/elaborative processing and perfunctory/transient processing as a dichotomy. We selected this “either/or” approach to provide straightforward exposition that allowed for competing research hypotheses. Moreover, the dichotomy conceptualization builds on Searle’s [93] philosophical distinction between prior intentions and intentions-in-action, as well as Kvavilashvili and colleagues’ [18] empirical isolation of implicit intentions. Nevertheless, when considering the Dynamic Multiprocess Framework’s proposal that bottom-up and top-down processes are both engaged for individual intentions [59], it may be more realistic (albeit less parsimonious) to expect that every time one encodes an intention that some aspects of encoding will be perfunctory (e.g., specific cues related to an overarching intention) and other aspects of encoding will be strategic/elaborative (e.g., the sequence of planned actions). If we conceptualize strategic/elaborative and perfunctory/transient encodings as part of a continuum, then the summed degree of strategic/elaborative processing likely depends on whether the intention is self-generated or other-generated [94], whether the content is important and complex [58], and whether the retrieval context is predictable and controllable [75]. Mapping the degrees of strategic-to-perfunctory processing during individual encodings seems a worthy, albeit challenging, goal for future research.

### Practical implications

From a translational perspective, our findings emphasize the importance of specifically encoding intentions [75]. Implementation intention encoding [86] is one strategy to improve goal fulfillment via re-phrasing a general intention into specific exemplars. For example, instead of “I need to get gas” one might state “When I see *the red gas station sign*, then I will remember to fill up my car with gas.” We previously found that implementation intention encoding increased the number of specific exemplars generated during a category prospective memory task, particularly when a structured “When…then” statement was paired with visual imagery of the intention [24]. Thus, even though specific exemplar encoding can occur via perfunctory processes, it can also be stimulated strategically via an implementation intention strategy. Increasing the probability of spontaneous retrievals via encouraging specific exemplar generation is likely to be one mechanism by which implementation intentions improve remembering of laboratory and naturalistic prospective memory tasks [95,96].

### Summary

Some prospective memory research has indicated that strategic/elaborative encoding, a view adapted from theories of planning [79], is required to successfully encode an intention [19,26,28]. The results of other prospective memory studies, however, indicate that aspects of encoding can be perfunctory/transient [18,29,30]. Our findings of the commonality of mind wandering, brief encoding durations, similarities across young and healthy older adults, and null associations between mind wandering and prospective memory performance, converge with the perfunctory view. In other words, some prospective memory encoding may be done “in passing.”
